# Impacts of Horticultural Mineral Oils and Two Insecticide Practices on Population Fluctuation of *Diaphorina citri* and Spread of Huanglongbing in a Citrus Orchard in Sarawak

**DOI:** 10.1100/2012/651416

**Published:** 2012-05-02

**Authors:** Stephen Chan Teck Leong, Fatimah Abang, Andrew Beattie, Roland Jui Heng Kueh, Sing King Wong

**Affiliations:** ^1^Faculty of Agriculture and Food Sciences, Universiti Putra Malaysia Campus Bintulu Sarawak, Jalan Nyabau, 97008 Bintulu, Sarawak, Malaysia; ^2^Faculty of Resource Science and Technology, Universiti Malaysia Sarawak, 94300 Kota Samarahan, Sarawak, Malaysia; ^3^Centre for Plant and Food Science, University of Western Sydney, Locked Bag 1797, Penrith New South Wales 2751, Australia

## Abstract

Aspects of the incidence and spread of the citrus disease huanglongbing (HLB) in relation to the vector *Diaphorina citri* population fluctuation were studied from January 1999 to December 2001 seasons in a 0.8 ha citrus orchard at Jemukan (1° 33′N, 110° 41′E), Southwest Sarawak in Malaysia. In relation to insecticide and horticultural mineral oils (HMOs) use, levels of HLB infection rose quite rapidly over the next 3 years in the unsprayed control and less rapidly in the other treatments such as imidacloprid, *n*C24HMO, and triazophos/cypermethrin/chlorpyrifos. Levels of HLB as determined by Polymerase Chain Reaction (PCR) were 42.2%, 9.4%, 11.4%, and 22.7%, respectively. The effects of *n*C_24_HMO and conventional pesticides on the citrus psyllid population and parasitoids in citrus orchard were also determined.

## 1. Introduction

The Asiatic citrus psyllid, *Diaphorina citri* Kuwayama (Hemiptera: Psyllidae), is widely distributed in the Oriental realm [1]. *D. citri* achieved major pest status when it was identified as the vector of Huanglongbing (HLB) or citrus greening disease (CGD) caused by *Candidatus Liberibacter asiaticus* (*α*-Protobacteria) [2] in Asia [3–8]. The distribution of *D. citri* is wider than that of the citrus greening bacterium, the major pathogen which transmits *D. citri* is very prolific and efficient vector of HLB [9], and its activity is the key to HLB spread. HLB is one of the most serious and devastating disease of citriculture in the world [7, 8, 10, 11], having destroyed around 50 million citrus trees since it was observed in China by Reinking [12], with most of these losses occurring in Southeast Asia [13]. HLB is currently found in China, Taiwan, Thailand, Vietnam, the Philippines, Indonesia, New Guinea; Japan and other major citrus growing areas in South Africa, USA, India, Pakistan, and Reunion Island, Brazil [14–20]. It is the greatest hindrance to the commercial development of viable citrus industries in tropical and subtropical Asia [21]. As a consequence, the income of farmers and national economies are seriously affected. 

In Malaysia, *D. citri* was first recorded in Ringlet, Cameron Highland and Ulu Tiram, Johor in 1978 [22], and symptoms of HLB were first noticed in Malaysia in the 1970s [23, 24], and its presence was confirmed in 1989 [25]. HLB together with its vector is now widely distributed in the lowlands and has been recorded in many parts of the country [26]. It has spread rapidly and gained national importance owing to the dramatic spread and transmission of HLB between 1989 and 1992 in Malaysia, which devastated citrus orchards in Peninsular and East Malaysia, including Sarawak [26, 27]. The once flourishing citrus industry in the Samarahan Division of Southwest Sarawak in Malaysia was completely destroyed by HLB in 1992 [27]. By 1991, the disease had destroyed a total area of 1,143 ha with more than 309,500 trees resulting in an estimated yield loss of more than 6,500 metric tons of fruit and an economic loss of RM 6.5 million [27, 28]. HLB and its vectors pose a major threat to the Malaysian citrus and nursery industries, especially in citrus rehabilitation. Furthermore, HLB is a highly destructive and fastidious disease of citrus. It is the primary cause of losses in citrus production in Asia [14], South Africa, and USA [20], and there is no successful treatment of infected trees. The development of sustainable management practices for minimizing the impact of HLB and its vector control options include the use of mineral oils, imidaclorprid, removal of alternate host plants for the disease and vector*-*near citrus orchard, removal of diseased trees, introduction of trees as wind breakers, rotation plantings and interplanting with a nonhost such as guava (*Psidium guajava*) can effectively reduce the incidence of *D. citri* and HLB in citrus orchards has been reported by some researchers [29–31]. Current controls of HLB in Sarawak are limited to the use of disease-free propagating stock, elimination of infected trees, and chemical control of* D. citri*. Ko [23] mentioned that sprays were required at least fortnightly in Malaysian orchards for control of *D. citri*, and more frequent applications were required during periods of flush growth. Current management strategies in Brazil involve annual application of some 8–18 sprays to trees less than 4-year-old [32]. Some researchers such as Tiwari et al. [20] reported the emerging insecticide resistance problem for Asian citrus psyllid if effective resistance management is not practiced. HLB is a devastating disease and not curable at present with chemical control. As such an integrated disease and vector management must be implemented. The studies on transmission, spread, and control of the HLB vector are relevant to the severity and urgency of the problem to the citrus and nursery industries in Malaysia and other major citrus growing areas. In this study, we report the incidence and spread of HLB in relation to the development and dispersal of *D. citri* population and their possible vector control in a citrus orchard.

## 2. Materials and Methods

### 2.1. Incidence and Spread of *D. citri* Population in a Citrus Orchard

Field studies conducted from January 1999 to December 2001 in a 0.8 ha citrus orchard at Jemukan (1° 33′N, 110° 41′E), Samarahan Division, Southwest Sarawak in Malaysia were focused on seasonal population changes, migration, and dispersal of *D. citri* as to detect changes in the incidence of vector and spread of citrus HLB in the citrus orchard and the impacts of the pesticides and horticultural mineral oils on the vector and its primary parasites. It comprised 200 initially disease-free, bud-grafted, honey mandarin plants (*Citrus* x *C. aurantium *Linnaeus) [Rutaceae: Aurantioideae] planted in 1999. Disease-free trees were used to establish the citrus orchard in which the study was based.

A total of 100 flushes were selected on each sampling date. Visual counts were taken on the number of psyllids (eggs, nymph, and adults) on the five twigs (each 10 to 20 cm long) per tree. All young shoot samples were about 6 to 10 cm long and had 5 immature leaves within the size range 2 to 4 cm long. Insect count and number of trees infested by psyllids were recorded weekly throughout citrus growing season.

### 2.2. Disease Incidence and Spread of HBL in relation to Psyllid Population

Incidence of HLB was monitored by visual inspection for signs of characteristic symptoms such as yellowing shoots, leaf mottling, and blotching. The number of HLB suspected trees in the orchard based on the visual symptoms was calculated monthly. Disease incidence was calculated as the number of trees expressing symptoms divided by the total number of trees in the orchard. Monthly samples of leaf midveins were collected on presumably infected trees, and field diagnosis was subsequently confirmed on the diseased trees. A molecular diagnostic technique, Polymerase chain reaction (PCR) procedures that amplify rDNAs fragments using HLB-specific primer 16S rDNA was used to confirm the presence of the bacterium in diseased trees.

### 2.3. Effects of Horticultural Mineral Oils and Conventional Pesticides on Oviposition, Nymphs, and Adult Mortality

The pesticide efficacy experiment had 4 treatments with 4 replicates arranged in a randomized complete block design. Each replicate comprised of 12 trees, and assessments were based on 6 central trees in each replicate. Other trees in each plot were used as spray drift buffers. The 4 treatments were (a) a control, unsprayed, (b) *n*C24 horticultural mineral oils (HMOs) (Ampol D-C Tron Plus: Ampol Rural, Sydney, Australia) applied as 0.35% v/v aqueous emulsions, (c) a conventional synthetic pesticide based on applications of triazophos (0.03%) (Hostathion 40EC, Aventis Crop Science), alternated with cypermethrin (0.125%) (Dow Agro Science) and chlorpyrifos (0.0125%), and (d) applications of a systemic neonicotinoid insecticide imidaclorprid (0.01%) (Confidor 200EC, Bayer, Selangor Darul Ehsan, Malaysia).

A motorised Fuji FP-25 knapsack sprayer was used to apply HMO at 0.35% (v/v) thoroughly to run off every 6 to 7 days and particularly during the annual flushing periods occurred in February-March, July, and September-October and the volumes applied per tree increased as the trees grew. The specifications of the HMO were given by Rae et al. [33]. All other pesticides were applied biweekly at the manufacturers' recommended rates.

### 2.4. Statistical Analysis

Data was subjected to one-way analysis of variance (ANOVA). The NCSS computer package was used for the ANOVA. For significant *F* values, the differences between the means were separated using the Fisher's least significant difference test at *P* ≤ 0.05.

## 3. Results and Discussion

### 3.1. Incidence and Spread of *D. citri* Population in a Citrus Orchard

**Figure 1:**
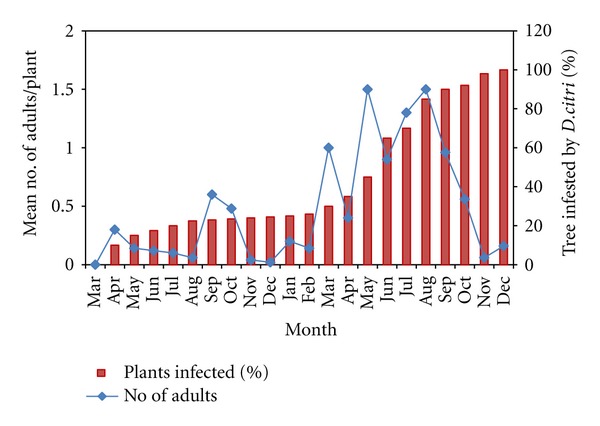
Incidence and spread of *D. citri* population in a citrus orchard at Jemukan.

Figure 1 indicates that a higher percentage of untreated trees are related to a higher numbers of adult psyllids. The value increased from 1.5% of trees infested to 46.0% one year after the first infestation was observed, and this value had jumped to 90% of trees infested 5-6 months later. It took about one year and nine months to infest all trees in the untreated citrus orchard. There was a linear increase in the number of trees becoming infested relative to time (*r* = 0.983, *P* < 0.001), and migration/dispersal of the psyllid population was mainly related to the main flush cycles occurred in February-March and September-October. The spread of *D. citri* from tree to tree throughout the growing seasons will result in the increase in the number of trees became infested in a citrus orchard as shown in Figure 1. The rapid build-up in *D. citri* population resulted from the exponential population growth and migration or dispersal of the psyllid population was mainly related to main flush cycles. This clearly indicates that the build-up in* D. citri *population was greatly influenced by the young flush growth, and the increase in populations of nymphs and eggs was greater than the adults in the citrus trees. This implies that a higher nymph numbers will become adults and subsequently result in a high adult population. The rate of infestation from tree to tree was not calculated, but Samways and Manicom [34] found that when *T. erytreae* invaded an orchard and its populations increased exponentially, the rate new trees became infected increased by 4% per day in an unsprayed orchard.

### 3.2. Disease Incidence and Spread of HBL in relation to Psyllid Population

**Figure 2:**
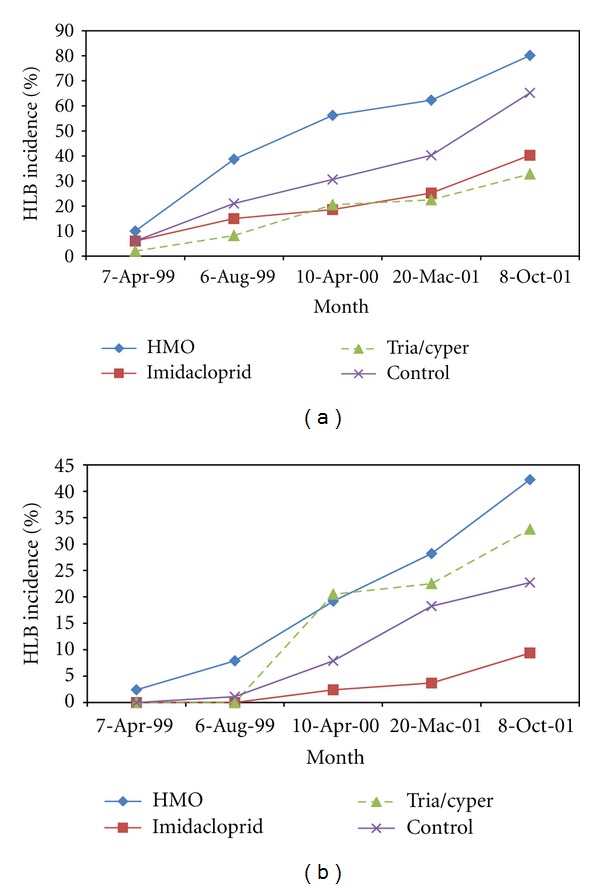
(a) Percentage of HLB-symptomatic infected trees on control and treated citrus orchard at Jemukan. (b) Percentage of HLB diseased trees on control and treated citrus orchard at Jemukan.

Figures 2(a) and 2(b) show percentage of HLB incidence on control and treated trees in citrus orchard. The percentage of infected trees increased progressively from 10% on 7th April 1999 to 38.7% on 6th August 1999 in the untreated control plots; only 2.4% of these plants gave positive PCR results (7th April 1999). By 10th April 2000, 19.2% out of the 56.2% of plants with symptoms of mild mottling and small leaves pointing upright gave positive PCR results. By October 2001, 80.2% of trees in the study orchard exhibited typical visual symptoms of the disease; within four years after planting 42.2% of trees in the unsprayed plots were infected (Figures 2(a) and 2(b)). The increase in incidence and spread of HLB in the citrus orchard was because of the high vector populations due to migration and/or dispersal of infective adults and their spread from infected trees to healthy trees during the flushing periods (February-March, July and September-October). The imidaclorprid and HMO-treated plots produced a lower percentage (9.4% and 11.4%) of diseased plants as compared with 22.7% in triazophos/cypermethrin/chlorpyrifos-treated plots (Figure 2(b)). The HMO treatment produced a lower percentage (11.4%) of diseased plants as compared with 42.2% in untreated control trees. The percentage of infested trees was greatly reduced in the sprayed plots as compared with unsprayed plots. This may attribute to the impact of HMO on the feeding behaviour of the psyllids and suffocation of nymphs and reduced oviposition due to behavioural effects on adult females and spray may have caused significantly adult mortality. Ingress of HLB was slower than untreated control in the experiment with imidacloprid reported by Gatineau et al. [35], in which the levels of HLB infection (symptomatic and PCR+) reached 24% and 74%, respectively, within 2 years and 96% in the untreated control. In China, citrus HLB often spread quickly in young citrus orchards; 50–70% of the citrus trees were infected before fruit production [36].

Certain mineral oils are known to reduce aphid colonization on plants, and thus the transmission of virus disease [37]. Vandenveken [38] further suggested that the oils might modify the charge of the stylet, thus impeding adsorption or elusion of virus particles, or the inhibitory properties of oils would hamper the exchange of charge between virus particles, aphid mouthparts, and plant cells. Clearly, the vector control programs practiced in this orchard did not prevent establishment and spread of HLB. The spread of disease in the *D. citri*-infested citrus orchard or transmission appears to be related to high vector populations and extensive inoculum reservoir [38]. Psyllid movement is highest when host plants are flushing and *T. erytreae* is reported to be highly infective on young flush [39]. Therefore, the prolong flushing during the flush cycles of young trees made them very attractive to the vector, and this can partly explain the rapid spread of disease in the psyllid infested trees within the citrus orchard. Schwarz et al. [40] showed a positive correlation between the degree of greening infection, the number of psyllids, and the rate of transmission. Psyllids are strongly attracted by yellow green of wavelength 550 nm [41], and making the diseased trees attractive target and thereby increasing the proportion of disease-carrying insects. Koizumi et al. [42] demonstrated that field collected psyllids were able to transmit HLB at a rate of at least 41% after 2 days of feeding. DNA hybridisation revealed that the proportions of viruliferous psyllids found in the infested citrus orchard in May and September ranged from 5% to 39%, respectively, in Sarawak [4]. These facts suggest that elimination of the pathogen is not possible in the absence of effective programs for removing diseased trees. Obviously *D. citri* was responsible for the rapid spread of HLB from trees to trees over time within the orchard. Gibson et al. [43] and Rice et al. [44] suggested that the insecticides with highly selective activity on insect and its natural enemies used to inhibit virus disease transmission should cause rapid know-down and prolonged incapacitation of the insect. Applying insecticides at critical flushing periods may be effective to some degree in reducing spread of disease and may require regular monitoring of citrus in order for time management strategies such as insecticide applications.

### 3.3. Effects of HMO and Conventional Pesticides on Oviposition, Nymphs, and Adult Mortality

**Figure 3:**
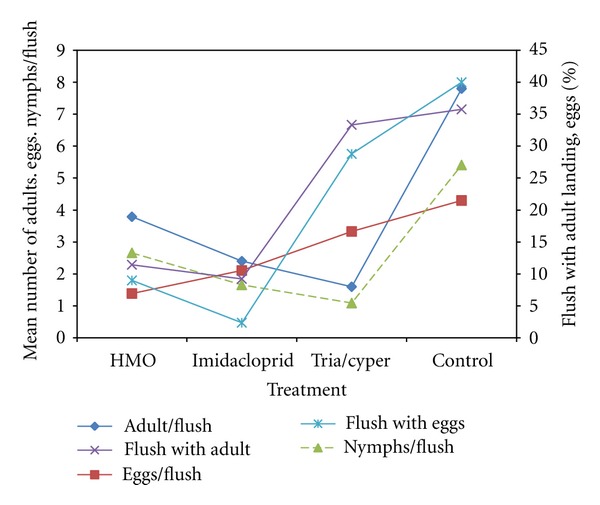
Effects of treatments on *D. citri* infestations on flush growth during August 2000.

The number of female psyllids landing on flushes differed significantly between treatments (*P* ≤ 0.01), but there were no differences between the unsprayed control and the triazophos/cypermethrin/chlorpyrifos treatment, or between the imidaclorprid and HMOs treatments (Figure 3). For eggs, there was a significant difference between treatments (*P* ≤ 0.01) for the percentage of flush on which eggs were laid; all spray treatments significantly reduced the number of flushes on which eggs were laid. Although more eggs were laid on unsprayed flushes than in the HMO treatment, there was no significant difference between treatments for the number of eggs laid per flush (Figure 3).

The mean number of nymphs and adults per flush was significantly reduced by all treatments (Figure 3). The mean number of nymphs and adults per flush was significantly reduced by all treatments (Figure 3). The HMO and imidaclorprid treatments were the most effective treatments for preventing adult females from landing on flushes (Figure 3). This outcome may have important implications for transmission of HLB, as it suggests that in these treatments the level and extent of feeding by adults would have been significantly less than the triazophos/cypermethrin/chlorpyrifos and unsprayed control treatment, and therefore transmission of the disease would have been less. All treatments did not affect the landing of female psyllids on sprayed flushes. Since the percentage of flushes landed on by female psyllids and percentage of flushes on which eggs were deposited in the HMO and imidaclorprid treatments were significantly lower than in the triazophos/cypermethrin/chlorpyrifos and unsprayed treatments (Figure 3), and because female psyllids were equally likely to land on sprayed and unsprayed flushes, the deposition of fewer eggs on sprayed flushes indicates that HMO and imidaclorprid sprayed flushes were discriminated against. This study clearly demonstrates that HMO and imidaclorprid reduced the number of eggs deposited by female psyllids and are presumably the result of oviposition deterrence. Oviposition deterrence as a result of oil deposits has also been demonstrated with Asiatic citrus psyllid [29, 30, 45], citrus leaf miner [33], codling moth, and whiteflies. The impact of the HMO on *D. citri* (see Figure 3) was attributed to suffocation of nymphs and reduced oviposition due to behavioural effects on adult females. Sprays may also have caused significantly adult mortality. The effect of imidaclorprid was higher than HMO in reducing the female psyllids per flush and the percentage of flush with eggs deposited on sprayed flushes probably because it is a systemic neonicotinoid insecticide and acts as a contact and stomach poison against psyllid.

### 3.4. Effects of HMO and Conventional Pesticides on Field Parasitism of *D. citri* Nymphs

**Figure 4:**
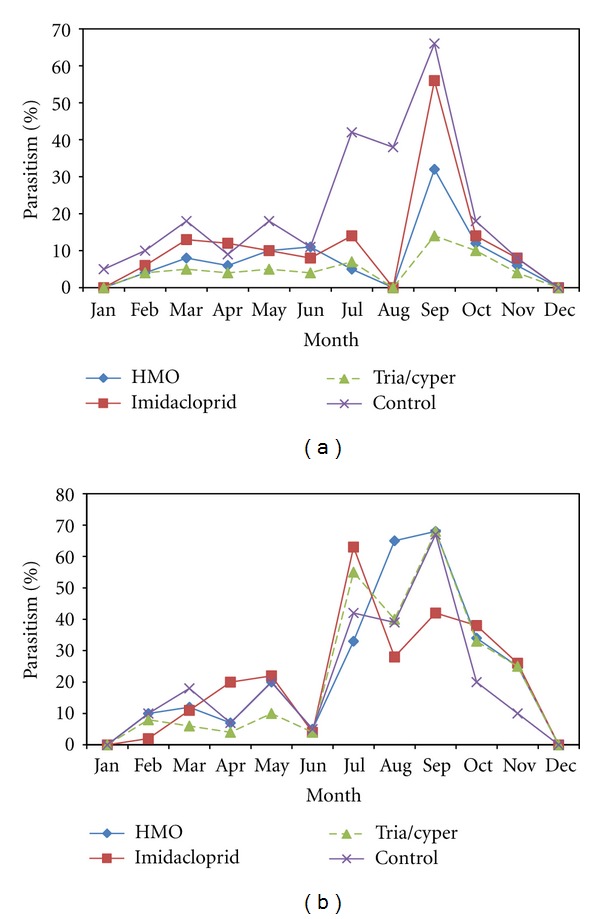
(a) Effect of treatments on percentage of parasitism on *D. citri* nymphs by *T. radiata* in a citrus orchard. (b) Effect of treatments on percentage of parasitism on *D. citri* nymphs by *D. aligarhensis* in a citrus orchard.

Regular field application of insecticides can directly reduce the number of *D. citri *nymphs available for parasitism. Despite the applications of insecticides treatments, the two primary parasitoids *Tamarixia radiata* (Waterston) [Hymenoptera: Eulophidae] or *Diaphorencyrtus aligarhensis *[46] [Hymenoptera: Encyrtidae] were still able to parasitise *D. citr*i nymphs and complete their development. This implies that if an insecticide spray fails to kill all *D. citri* nymphs, then either parasitoid may be able to attack the survivors. Good potential therefore exists for that use of more selective insecticide, for example, HMO and imidaclorprid in conjunction with the parasitoids to manage *D. citri* population more effectively. The results (Figures 4(a) and 4(b)) of this study showed that HMO appeared to have less impact on both primary parasitoids than synthetic insecticides. The parasitism rate might be slightly reduced because as parasitoids have to search for hosts within canopies, they inevitably come into contact with sprayed leaf surfaces. Further as a result of the psyllid numbers being reduced more time is spent searching for psyllid. The results suggest an important role for both *T. radiata* and *D. aligarhensis,* the two parasitoids in IPM programs for *D. citri*, particularly those based on selective products such as HMO and imidaclorprid. In summary, the results showed that both imidaclorprid and HMO could be used as key components in integrated pest and disease management program for sustainable control of *D. citri*. Further research may be warranted to determine if the number of sprays required annually can be minimised in relation to the phenologies of *D. citri *and its plant hosts.

## 4. Conclusion

There was a linear increase in the number of trees becoming infested relative to time within the citrus orchard, and migration and dispersal of the adults was also related to flushing cycles. The HMO and imidaclorprid treatments were the most effective treatments for preventing adult females from landing on flushes. The percentage of infested trees was greatly reduced in the sprayed plots as compared with unsprayed plots. There is an important role for both *T. radiata* and *D. aligarhensis,* in IPM programs for *D. citri*, particularly those based on selective products such as HMO and imidaclorprid.

As for all insect-borne diseases, the aim is to reduce vector populations and their contact with infected host plants. In tropical humid areas like Malaysia, control of *D. citri* population requires the use of pesticides, particularly in time when conditions favour its development. The prevention of high psyllid populations especially during the flushing cycle is extremely important in checking the spread of the disease. Thus, insecticide sprays must target at these young growth flushes. It is very important to protect citrus trees from HLB infection when they are young. The focus on killing the psyllids in order to limit the spread of HLB has led to emphasis being placed on reducing their impact by minimizing feeding by adults and oviposition.

A successful IPM programme for HLB and its vector in individual orchard is achieved by combining proper nursery management with early eradication of all infected and uneconomic trees, use of healthy planting materials, and effective control of psyllid vector by judicious insecticidal spray schedule supplemented with natural enemies.
